# Adolescent-onset primary small cell neuroendocrine carcinoma of the nasal cavity: a rare and aggressive entity with Favorable outcome

**DOI:** 10.1093/omcr/omag055

**Published:** 2026-05-10

**Authors:** Imane Boujguenna, Mohamed Amine Haouane, Ismail Aissaa, Hind Boujguenna, Soufiane Abdouh, Abdelwahab Dallouri, Hiba Daoudi, Mohamed Ali Elallami, Aya Elmoudenib, Wissal Biad, Arwa Azgaoun, Fatima Boukis

**Affiliations:** Guelmim Faculty of Medicine and Pharmacy, Ibn Zohr Agadir University Guelmim 81000, Morocco; Department of Pathology, Caddi Ayyad University of Marrakesh/Ibn Sina Military Hospital, Marrakesh 40000, Morocco; Moulay Elhassan Military Hospital, Department of Anesthesiology and Critical Care, Guelmim 81000, Morocco; Private University of Marrakesh, Marrakesh 40000, Morocco; Private Practice Physician, Marrakesh 40000, Morocco; Guelmim Faculty of Medicine and Pharmacy, Ibn Zohr Agadir University Guelmim 81000, Morocco; Guelmim Faculty of Medicine and Pharmacy, Ibn Zohr Agadir University Guelmim 81000, Morocco; Guelmim Faculty of Medicine and Pharmacy, Ibn Zohr Agadir University Guelmim 81000, Morocco; Guelmim Faculty of Medicine and Pharmacy, Ibn Zohr Agadir University Guelmim 81000, Morocco; Guelmim Faculty of Medicine and Pharmacy, Ibn Zohr Agadir University Guelmim 81000, Morocco; Guelmim Faculty of Medicine and Pharmacy, Ibn Zohr Agadir University Guelmim 81000, Morocco; Al AMAL Pathological Anatomy Laboratory, Guelmim 81000, Morocco

**Keywords:** small cell neuroendocrine carcinoma, nasal cavity, adolescent, chemoradiotherapy, sinonasal tumors, rare tumors, pediatric oncology

## Abstract

Small cell neuroendocrine carcinoma (SCNEC) of the nasal cavity is extremely rare and aggressive, particularly in pediatric and adolescent patients. We report the case of a 17-year-old Moroccan male presenting with intermittent unilateral nasal obstruction. Endoscopy revealed a vascular-appearing mass in the left nasal fossa, which was excised. Histopathological analysis supported by immunohistochemistry confirmed high-grade SCNEC, with a Ki-67 index of 90% and diffuse expression of chromogranin and synaptophysin. Imaging demonstrated no metastatic disease, and the tumor was staged as pT1N0M0 (Stage I). The patient subsequently underwent adjuvant chemoradiotherapy beyond the initial excisional biopsy. At 12 months of follow-up, no recurrence was detected. This case highlights the importance of early detection and illustrates that organ-preserving, multimodal non-surgical management can achieve disease control in early-stage nasal SCNEC in adolescents.

## Introduction

Primary small cell neuroendocrine carcinoma (SCNEC) of the nasal cavity is an extremely uncommon malignancy characterized by rapid proliferation, early recurrence, and poor prognosis. While typically diagnosed in adults, its occurrence in adolescents is exceptional, with only isolated cases documented [1, 2]. Due to the rarity of this presentation, clinical guidelines for diagnosis and treatment remain limited.

We present a case of early-stage SCNEC of the nasal cavity in a 17-year-old male and discuss diagnostic challenges, therapeutic decision-making, comparison with the existing pediatric and adolescent literature, and the relevance of organ-preserving multimodal therapy.

## Case presentation

A 17-year-old male with no significant medical history presented with several months of persistent left-sided nasal obstruction. Nasal endoscopy showed a reddish, vascular-appearing lesion occupying the left nasal cavity. Given the benign appearance, the initial differential diagnosis included inflammatory polyps and benign vascular tumors. The mass was completely excised via an endonasal approach; it measured approximately 1 cm.

**Figure 1:**
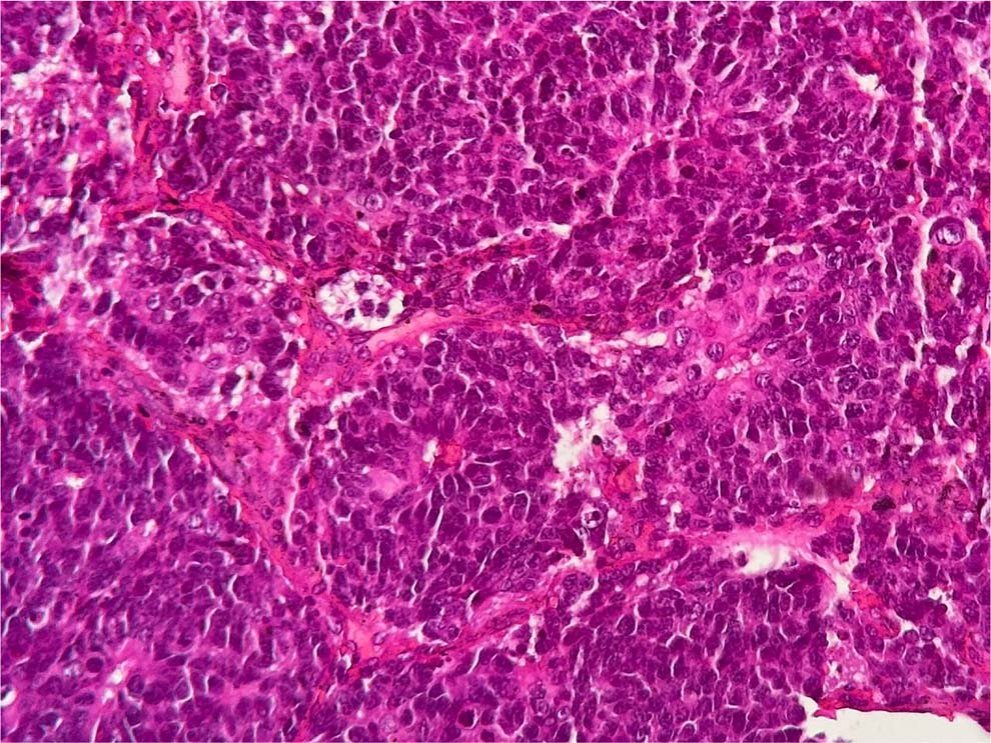
Hematoxylin–eosin staining showing a dense carcinomatous proliferation of small cells with hyperchromatic nuclei, scant cytoplasm, focal necrosis, and a high mitotic index.

**Figure 2:**
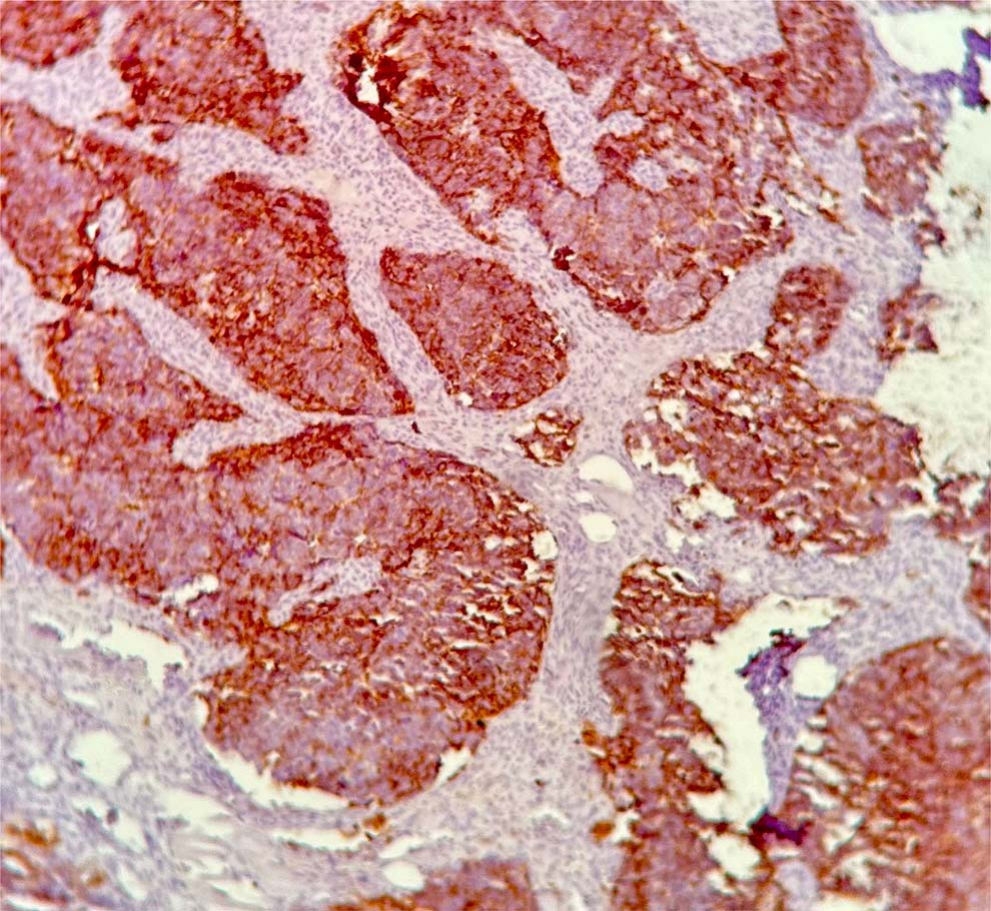
CD56 immunostaining demonstrating strong membranous positivity in tumor cells, supporting neuroendocrine differentiation.

**Figure 3:**
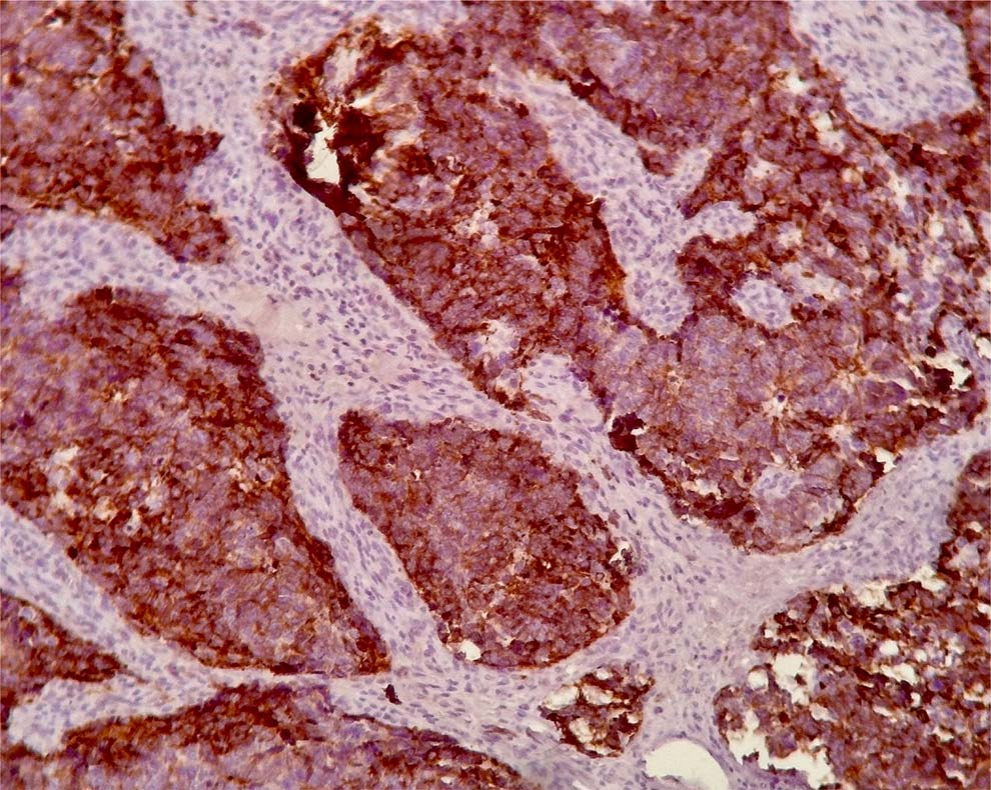
Synaptophysin immunostaining showing diffuse granular cytoplasmic positivity.

**Figure 4:**
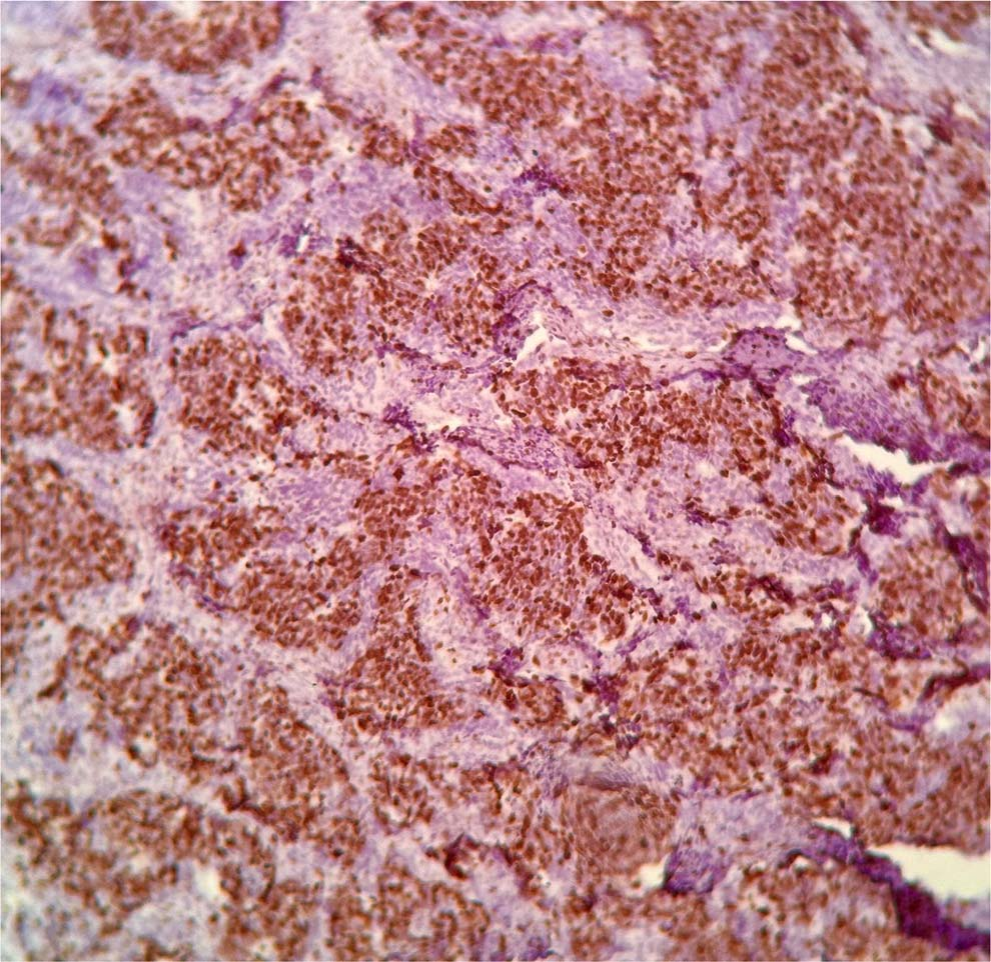
Ki-67 immunostaining revealing a high proliferation index (≈90%), consistent with high-grade SCNEC.

Histopathological examination demonstrated small to medium-sized tumor cells with hyperchromatic nuclei, scant cytoplasm, areas of necrosis, and high mitotic activity (>10 mitoses/2 mm^2^) (Fig. 1). Immunohistochemistry revealed strong membranous CD56 positivity (Fig. 2), diffuse synaptophysin (Fig. 3) and chromogranin expression, and a Ki-67 index of 90% (Fig. 4), confirming high-grade SCNEC.

Staging with cervicofacial MRI and thoraco-abdominopelvic CT scan showed no regional or distant spread. PET/CT was not performed because of the very small size and early stage of the tumor; however, it may be useful in more advanced lesions. Surgical margins were negative. The tumor was classified as pT1N0M0 (Stage I).

A multidisciplinary tumor board recommended adjuvant concurrent chemoradiotherapy. The patient received:

Cisplatin 75 mg/m^2^ on Day 1.

Etoposide 100 mg/m^2^ on Days 1–3.

Cycles every 21 days (4 cycles total).

Concomitant radiotherapy was delivered according to institutional protocol.

Monthly endoscopic evaluations and a 3-month MRI showed no recurrence. At 12 months, the patient remained disease-free with preserved sinonasal function and no treatment-related complications.

## Discussion

SCNEC of the nasal cavity is rare, and its occurrence in adolescents is extraordinary, with only isolated cases described [1, 2]. In previously reported pediatric and adolescent cases, tumors were frequently diagnosed at advanced stages, often requiring radical surgical resection followed by adjuvant chemoradiotherapy. Symptoms such as nasal obstruction or epistaxis commonly mimic benign sinonasal disease, contributing to delayed diagnosis [3, 4].

Compared with the literature, our case is notable for: one of the youngest patients described with nasal SCNEC; diagnosis at an early stage (pT1N0M0), whereas most cases in adolescents present later; successful disease control using organ-preserving multimodal therapy, rather than radical surgery.

This supports the growing evidence that early detection plays a decisive role in improving clinical outcomes.

### Diagnostic considerations

Definitive diagnosis relies on histopathology combined with neuroendocrine markers such as CD56, synaptophysin, and chromogranin A [5, 6]. A high Ki-67 index, as in our patient (90%), further supports a high-grade tumor.

### Treatment approach and evolving trends

Traditional management emphasizes surgical resection followed by radiotherapy [7]. However, studies have demonstrated radiosensitivity and chemosensitivity of SCNEC, supporting chemoradiotherapy as a primary or adjuvant treatment modality [8–10]. Organ-preserving approaches are increasingly favored in young patients to avoid functional morbidity.

Recent reports on head and neck neuroendocrine carcinomas highlight the variability in prognosis and the value of multimodal therapy [11, 12]. Our case aligns with these findings, illustrating that early-stage SCNEC may be effectively managed with adjuvant chemoradiotherapy after conservative excision.

### Prognosis in head and neck tumors

Recent evidence reinforces that survival outcomes for head and neck neuroendocrine tumors depend heavily on stage and treatment strategy [13, 14]. Our patient’s favorable short-term outcome reflects the importance of early-stage detection and the potential benefits of aggressive multimodal management.

### Limitation

A 12-month follow-up, although currently favorable, remains a short period for SCNEC, which may recur late. Long-term surveillance is necessary.

## Implications for health education practice

This case emphasizes the need for clinicians and trainees to consider rare neuroendocrine tumors in young patients presenting with persistent unilateral nasal symptoms. Educational efforts should encourage timely biopsy of suspicious lesions and promote multidisciplinary collaboration when managing rare tumors.

## Recommendations

Multicenter studies and prospective registries are needed to define optimal management strategies for nasal SCNEC and clarify prognostic markers in younger populations.
